# Variations in Theta/Beta Ratio and Cognitive Performance in Subpopulations of Subjects with ADHD Symptoms: Towards Neuropsychological Profiling for Patient Subgrouping

**DOI:** 10.3390/jpm13091361

**Published:** 2023-09-07

**Authors:** Wendy Verónica Herrera-Morales, Julián Valeriano Reyes-López, Karen Nicte-Ha Tuz-Castellanos, Desiree Ortegón-Abud, Leticia Ramírez-Lugo, Efraín Santiago-Rodríguez, Luis Núñez-Jaramillo

**Affiliations:** 1 Laboratorio de Neurofisiología, Departamento de Ciencias Médicas, División de Ciencias de la Salud, Universidad Autónoma del Estado de Quintana Roo, Chetumal 77039, Mexico; wendyhm@uqroo.edu.mx (W.V.H.-M.); castellanosk32@gmail.com (K.N.-H.T.-C.); 2 Unidad de Neurodiagnóstico y Rehabilitación “Dr. Moisés López Gonzáles” Secretaria de Vinculación y Servicios Universitarios, Facultad de Ciencias Naturales, Universidad Autónoma de Querétaro, Querétaro 76000, Mexico; opdeih@yahoo.com; 3 Universidad Santander, Montañas Rocallosas 409, Lomas de Chapultepec, Ciudad de México 11000, Mexico; desiree.ort.abu@gmail.com; 4 División de Neurociencias, Instituto de Fisiología Celular, Universidad Nacional Autónoma de México, Ciudad de México 04510, Mexico; letyrlugo@gmail.com; 5 Diagnóstico, Tratamiento e Investigación Neurológica, S.C. Querétaro, Santiago de Queretaro 76177, Mexico; efmx2000@yahoo.com.mx

**Keywords:** ADHD, cognitive performance, qEEG, patients subgrouping

## Abstract

ADHD is a neurodevelopmental disorder appearing in childhood but remaining in many cases in adults. There are both pharmacological and non-pharmacological approaches to treating ADHD, but they do not have the same efficacy in all subjects. Better knowledge of the neurophysiological basis of this disorder will allow for the design of more effective treatments. Studies performing qEEG analysis in children suggest the existence of subgroups of ADHD patients with different neurophysiological traits. There are fewer studies in adults, who might have undergone plastic changes allowing them to cope with ADHD symptoms along with brain maturation. Herein, we study cognitive performance and the theta/beta ratio in young adults with ADHD symptoms. We found that subjects with ADHD symptoms and low working memory performance (*n* = 30) present higher theta/beta ratios than controls (*n* = 40) at O2 and T6 in the eyes-closed condition, as well as a tendency toward a higher theta/beta ratio at O1 and Cz. Subjects with ADHD and high working memory performance (*n* = 50) do not differ from the controls in their theta/beta ratios at any derivation. Our results suggest that neuropsychological profiling could be useful for patient subgrouping. Further research will allow for the distinction of neuropsychological profiles and their neurophysiological correlates, leading to a better classification of ADHD subtypes, thus improving treatment selection.

## 1. Introduction

ADHD is a neurodevelopmental disorder presenting with hyperactivity, impulsivity and inattention symptoms. It is diagnosed mainly in children, although it is also present in adults [1,2,3].

There are different therapeutic approaches to treating ADHD patients, including both pharmacological and non-pharmacological treatments. The literature supports the efficacy of different drugs [4,5,6] in treating ADHD symptoms. Similarly, there are reports showing a good effect of different non-pharmacological approaches, such as behavioral parent training [7], cognitive behavioral therapy [8,9] and neurofeedback [10,11], to reduce ADHD symptoms. However, it is also clear that the effect of these treatments is not the same in all cases. For example, while some patients benefit from pharmacological treatment with stimulant or non-stimulant drugs, there are also patients who do not respond to these treatments [12,13,14,15]. Similar results are observed with non-pharmacological treatments, for example, with neurofeedback. While some meta-analyses conclude that neurofeedback produces an important reduction in symptoms [16,17,18], other studies do not find a robust effect [19], or find an effect only when neurofeedback is combined with pharmacological treatment [20]. The reasons for these differences are not fully understood, but they might be the result of the diverse etiology of this disorder, which could lead to various possible neurophysiological correlates of ADHD, albeit all producing similar symptomatology that fit the diagnostic criteria for ADHD [21].

As with any other disorder, the development of better therapeutic strategies for ADHD depends on an improved understanding of the underlying physiological changes associated with the presence of its symptoms. In this regard, different studies address the changes in brain structure and function associated with ADHD. A recent large-scale study in non-referred patients, with a high representativeness of the general population, analyzed magnetic resonance imaging in children with and without ADHD. They report reductions in the surface area of the anterior and posterior cingulate cortex, middle an inferior temporal gyri, precentral and postcentral gyri, lingual gyrus, pericalcarine cortex and cuneus [22].

Other studies have addressed, through quantitative electroencephalography (qEEG), the neurophysiological correlates of ADHD, mainly in children and adolescents. Many reports indicate that ADHD patients present an increase in slow-wave and a decrease in fast-wave activity [23,24]. A frequently used approach for the study of slow/fast waves in qEEG is the analysis of theta/beta ratios. Various studies report an increased theta/beta ratio in children and adolescents with ADHD [2]. However, this approach is not always optimal, and due to the heterogeneity found in the neurophysiological correlates of ADHD, some groups have proposed the idea of subgrouping ADHD patients according to their brain activity [25]. For example, a study performed on Thai children with ADHD reports different subgroups of patients considering the analysis of theta-relative power based on their brain mapping performance. The authors propose a relationship of the different qEEG profiles with specific neuropsychological profiles [26]. Similarly, another study separates children with ADHD into three subgroups based on their qEEG analysis. In another study, groups also differed in their scores in various tests that evaluate the presence of ADHD symptoms. However, they found no differences in their scores for other scales commonly used when evaluating children, such as depression, impulsiveness and anxiety tests, among others [27].

Studies in adults have failed to find the neurophysiological correlates described in children [28], including changes in theta/beta ratio [29]. The data that seem to be consistent in adults with ADHD is a decrease in gamma activity [30,31]. Since a large number of children with ADHD maintain this disorder as adults [32,33], it is important to determine the neurophysiological changes associated with this disorder in adults in order to improve the therapeutic approaches used in this age group. Furthermore, given the brain maturation process, as well as plastic changes associated with the development of strategies to cope with ADHD symptoms, as the patients grow older, the determination of neurophysiological correlates of ADHD in adults becomes more difficult.

Variations in neurophysiological correlates of the same condition might lead to differences in other parameters, such as cognitive performance, between subjects filling the same diagnostic criteria for a particular condition. For example, a study performed in alcohol-dependent subjects reported two different subgroups, each with different alterations in brain activity and different cognitive performance, albeit with the same diagnosis [34]. Thus, matching cognitive performance with specific qEEG correlates in persons with the same disorder might improve the sub-classification of patients with different alterations in brain activity.

Patients with ADHD represent a heterogeneous group, where persons filling the same diagnostic criteria might differ greatly in the etiology for this condition, as well as in its neurophysiological correlates [21]. Fitting such diversity into the same clinical criteria could be the reason for variations in the effects of different treatments, either pharmacological or non-pharmacological, on patients with the same diagnosis [12,13,14,15,16,19,20]. Since different subpopulations exist within patients diagnosed with ADHD, efforts directed toward identifying specific subgroups will help to achieve better treatment selection. Some attempts have been made by classifying children with ADHD based on qEEG analysis [26,27], while this approach has not been attempted in adults, whose neurophysiological correlates of ADHD differ from those of children [28,29]. Furthermore, different alterations in brain activity present in patients with ADHD could affect cognitive performance in different forms, so cognitive performance could help to distinguish between subpopulations of subjects with similar diagnoses. Altogether, the purpose of this work is to assess whether cognitive performance can be used to identify subgroups of persons with ADHD symptomatology presenting different neurophysiological correlates.

## 2. Materials and Methods

### 2.1. Participants

Participants were first-year health sciences students who were verbally invited to participate in this study during their classes. All procedures and data management were carried out in accordance with the Declaration of Helsinki, and the protocol was approved by the Committee of Evaluation of Research Functions of the Health Sciences Division. All participants signed an informed consent form. Participants reported no current or previous history of neurological illness and were not under any pharmacological treatment that could affect EEG activity.

### 2.2. Clinical Evaluation

Risk of ADHD was assessed through the Spanish version of the Adult ADHD Self-Report Scale (ASRS-V1.1), which has been previously validated in Spanish and Mexican populations [35,36]. The ASRS-V1.1 is used in other studies to determine the presence of ADHD symptoms [37,38,39]. It should be noted that the World Health Organization participated in creating the symptom checklist for this test.

Additionally, participants answered the Spanish version of the Alcohol Use Disorders Identification Test (AUDIT) to assess hazardous alcohol consumption and risk of alcohol dependence [40]. As previously reported by our group [41], we used an integrated interpretation based on two different approaches [40,42]. Participants also completed the Plutchik suicide risk test, Beck depression inventory and Barrat’s impulsiveness scale. These instruments were selected based on our previous studies [41,43].

### 2.3. Working Memory Test

Participants completed a working memory test using the software e-Prime 2.0. In each trial, participants were presented with a series of digits from 1 to 6 appearing on the screen. After the last digit of each series, the computer emitted a bleep sound and the test digit appeared in red, participants had to determine whether the test digit was present in the previously presented series. The working memory test consisted of 48 trials. Accuracy index and reaction time were recorded and extracted through the same software for statistical analysis.

### 2.4. EEG Recording

EEG recording was performed in a dimly lit room where participants were comfortably seated. Recording was performed in the closed-eyes condition using a 19-channel Medicaid Fenix electroencephalograph (Neuronic Mexicana S.A. de C.V., Mexico City, Mexico). Low- and high-frequency filters were set at 0.5 and 30 Hz, respectively, and a 50/60 Hz notch filter was used. Sampling frequency was 240 Hz, with a 16-bit resolution. Signal was collected through 19 electrodes placed in the international 10/20 system (FP1, FP2, F7, F8, F3, F4, T3, T4, C3, C4, T5, T6, P3, P4, O1, O2, FZ, CZ and PZ) fitted in an electrode cap (Electro-cap International, Inc., Eaton, OH, USA). Linked mastoids were used as references, and Fpz was used as the ground electrode. A clinical neurophysiologist (ES-R) performed rigorous visual analysis of all EEG recordings.

### 2.5. Quantitative Analysis of EEG

EEG segments of at least 2.56 s were manually selected until they amounted to at least one minute. The selection process included only segments where the subject was awake and presented alpha activity. We also excluded segments with muscular activity or blinking.

We used the Quantitative EEG Analysis software (Neuronic Mexicana S.A. de C.V., México) to obtain Absolute Power (AP) for the theta (3.91–7.42 Hz) and beta (12.89–19.14 Hz) frequency bands [44] by means of the fast Fourier transform algorithm. We applied subtraction of the global scale factor (GSF) to both AP measures to decrease non-physiological variability. For quantitative analysis, all EEG recordings were reformatted to the average reference [45]. We calculated theta/beta ratio for each derivation using the formula theta AP/beta AP.

### 2.6. Statistical Analysis

For comparison between the ADHD and control groups, we used unpaired T tests for theta/beta ratio on all 19 derivations, as well as for the accuracy index and reaction time values of the working memory test, and for the scores of Barrat’s impulsiveness scale. When analyzing ADHD subgroups, we performed ANOVAs and Fisher post hoc tests in all cases. All analyses were performed using Statview software (version 4.57).

## 3. Results

### 3.1. Subjects

After excluding subjects with EEG abnormalities, 207 subjects who completed all of the clinimetric tests remained. Data from all of the subjects rating positive for ADHD symptoms were considered for statistical analyses, while only those subjects rating negative for all the applied tests (ADHD, alcohol use disorders, depression and suicide risk) were included in the control group. Accordingly, 61 subjects rating negative for ADHD but positive for the other tests (alcohol use disorders, depression or suicide risk) were excluded from the analysis. After the exclusion of subjects who did not fit the inclusion criteria, only 146 subjects were included in the analysis, 66 rated negative for all the applied tests and were included in the control group, and 80 subjects rated positive for ADHD in the ASRS-V1.1. The gender and age composition for each group can be found in Table 1. Only 39 subjects with ADHD did not rate positive for the other tests, while 17 rated positive for hazardous alcohol consumption, 7 for alcohol dependency, 19 for depression and 25 for suicide risk. Some of the subjects rated positive for more than 1 comorbidity. This is in agreement with previous reports indicating the existence of comorbidities in ADHD patients [46,47].

Subjects within the ADHD group varied greatly in their performance on the working memory test, highlighting cognitive heterogeneity in these subjects. Given the existence of subgroups of ADHD patients reported in children [26,27], and considering that adults with ADHD symptoms might have undergone important plastic changes both associated with brain maturation and as a way of coping with ADHD symptoms, we assumed that adult subjects with ADHD symptoms might also represent a heterogeneous group. Furthermore, since previous attempts at defining subgroups of ADHD patients have addressed this task based on qEEG activity, it is reasonable to suppose that different neurophysiological correlates in persons with similar symptomatology might impact other areas such as cognitive performance. Thus, we performed two sets of statistical analyses, one comparing controls versus subjects with ADHD symptoms, and another separating ADHD subjects in two different groups based on their accuracy index (AI) in the working memory test, the first with an AI above 0.9 (ADHD HAI, *n* = 50) and the second with an AI ≤ 0.9 (ADHD LAI, *n* = 30). Since the controls greatly outnumbered both ADHD groups addressed in the second analysis, we randomized our controls using SPSS software to select only 40 controls. The age and gender composition of the groups for this analysis can be found in Table 2.

### 3.2. Analysis with Two Groups: ADHD and Controls

#### 3.2.1. Working Memory Test

**Figure 1:**
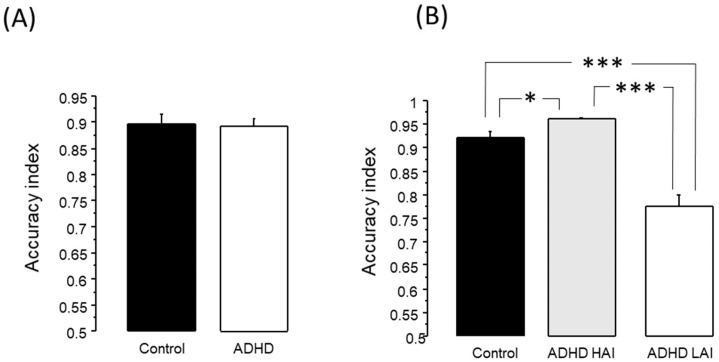
Accuracy index in the working memory test (mean + standard error). (**A**) Comparing controls versus all subjects with ADHD. (**B**) Comparing controls versus two subgroups of subjects with ADHD. * *p* < 0.05, *** *p* < 0.0001.

The unpaired T test revealed no difference between the ADHD and control groups, in either reaction time or accuracy index (Figure 1A).

#### 3.2.2. Analysis of Theta/Beta Ratio

**Figure 2:**
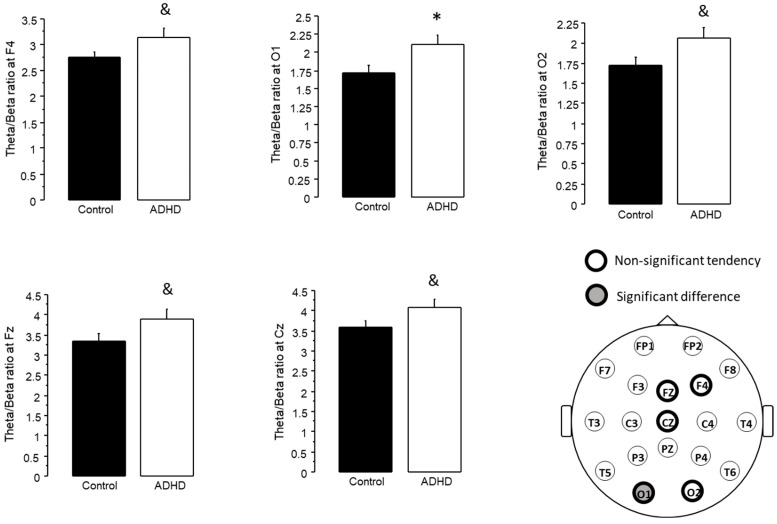
Sites where subjects with ADHD differed from controls in their theta/beta ratio (mean + standard error). * *p* < 0.05, & *p* < 0.1 (non-significant tendency).

The unpaired T test comparing the theta/beta ratio between the ADHD and control groups revealed that subjects with ADHD symptoms presented a higher theta/beta ratio than the controls at O1 (ADHD 2.105 ± 0.138, control 1.717 ± 0.102, *p* < 0.05). It should be noted that this tendency was also observed at F4 (ADHD 3.134 ± 0.179, control 2.745 ± 0.114), O2 (ADHD 2.063 ± 0.13, control 1.724 ± 0.104), Fz (ADHD 3.895 ± 0.246, control 3.363 ± 0.166) and Cz (ADHD 4.077 ± 0.223, control 3.579 ± 0.223), where the *p* value was lower than 0.1, but did not reach significance (*p* < 0.05) (Figure 2).

#### 3.2.3. Analysis of Impulsiveness Scores

**Figure 3:**
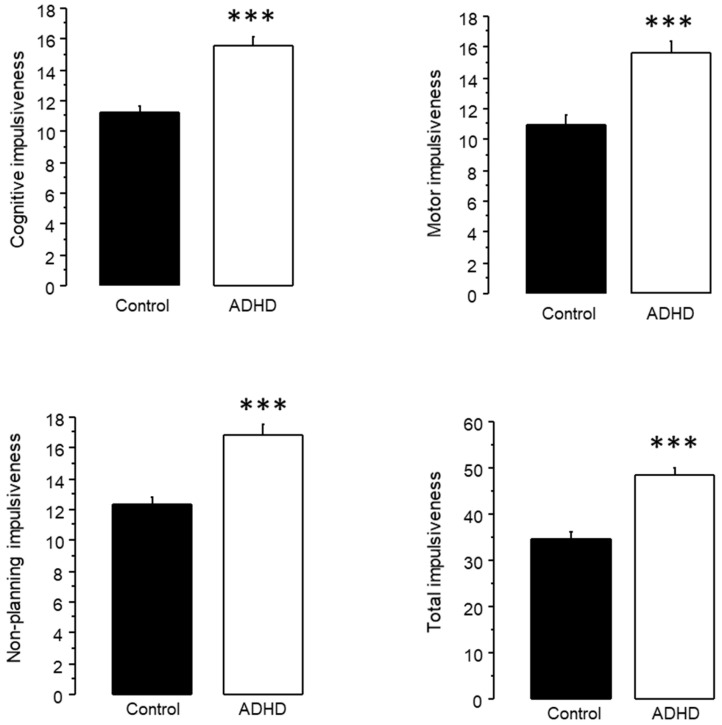
Impulsiveness in subjects with ADHD symptoms and controls (scores + standard error). *** *p* < 0.0001.

The unpaired T test revealed that subjects with ADHD symptomatology presented higher scores than the controls in cognitive (ADHD 15.588 ± 0.536, control 11.212 ± 0.442, *p* < 0.0001), motor (ADHD 15.688 ± 0.689, control 10.955 ± 0.674, *p* < 0.0001), non-planning (ADHD 16.837 ± 0.74, control 12.333 ± 0.548, *p* < 0.0001) and total impulsiveness (ADHD 48.362 ± 1.533, control 34.818 ± 1.329, *p* < 0.0001) (Figure 3).

### 3.3. Analysis with Three Groups: ADHD HAI, ADHD LAI and Controls

#### 3.3.1. Working Memory Test

As revealed by the ANOVA test, there was a significant effect of group on AI for the working memory test (F2,117 = 45.84, *p* < 0.0001). The Fisher post hoc test revealed that ADHD LAI presented lower AI (0.777 ± 0.024) than both the ADHD HAI (0.961 ± 0.004) and control (0.921 ± 0.014) groups (*p* < 0.0001 in both cases). Additionally, AHDH HAI presented higher AI than the control group (*p* < 0.05) (See Figure 1B).

We did not find any difference in reaction time.

#### 3.3.2. Analysis of Theta/Beta Ratio

**Figure 4:**
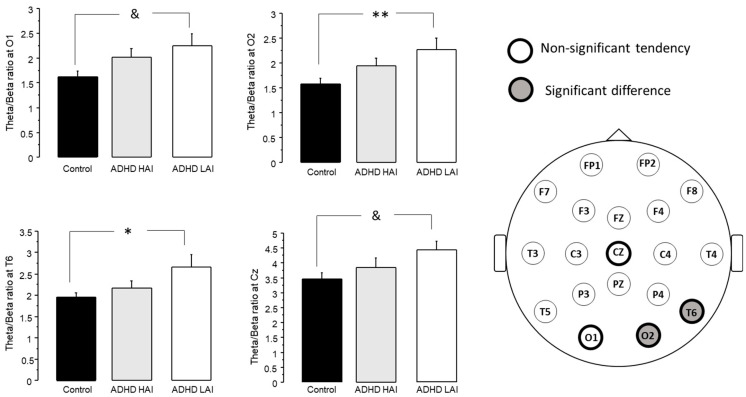
Sites where subjects with ADHD differed from controls in their theta/beta ratio (mean + standard error). * *p* < 0.05, ** *p* < 0.01, & *p* < 0.1 (non-significant tendency).

The ANOVA test revealed an effect of group on the theta/beta ratio at O2 (F2,117 = 3.934, *p* < 0.05) and T6 (F2,117 = 3.075, *p* < 0.05). The post hoc tests revealed a higher theta/beta ratio in ADHD LAI than in the controls at both O2 (ADHD LAI 2.273 ± 0.225, control 1.58 ± 0.11, *p* < 0.01) and T6 (ADHD 2.66 ± 0.293, control 1.952 ± 0.115, *p* < 0.05) (Figure 4).

Although no significant difference was reached in other derivations, a tendency towards an effect of group was observed at O1 (F2,117 = 3.071, *p* = 0.0501) and Cz (F2,117 = 2.69, *p* = 0.0721). Similarly, post hoc tests indicated a higher theta/beta ratio in the ADHD LAI group when compared with the controls at O1 (ADHD LAI 2.249 ± 0.229, control 1.622 ± 0.108, *p* < 0.05) and Cz (ADHD LAI 4.457 ± 0.294, control 3.467 ± 0.195, *p* < 0.05) (Figure 4).

#### 3.3.3. Analysis of Impulsiveness Scores

**Figure 5:**
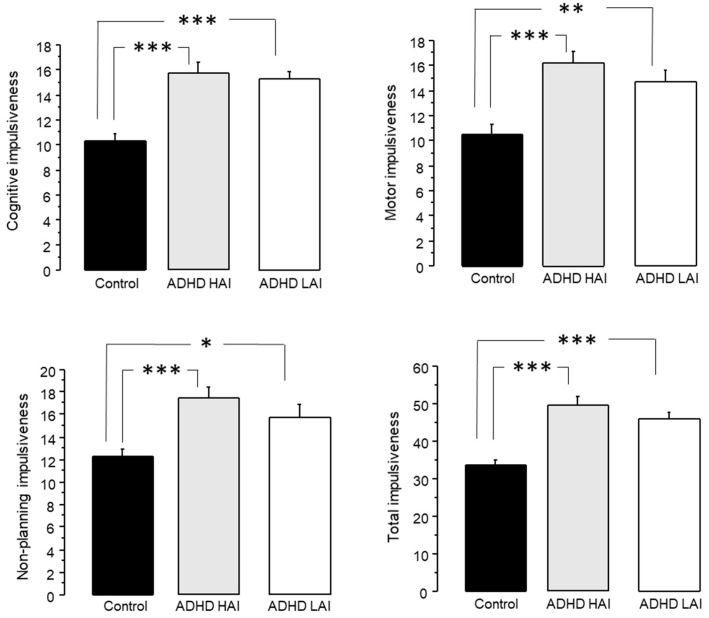
Impulsiveness scores in subjects with ADHD symptoms, either with high accuracy index (ADHD HAI) or low accuracy index (ADHD LAI) in the working memory test, and controls (scores + standard error). * *p* < 0.05, ** *p* < 0.01, *** *p* < 0.0001.

The analysis of cognitive impulsiveness through ANOVA revealed an effect of group (F2,117 = 19.323, *p* < 0.0001). The Fisher post hoc test indicated that both ADHD LAI (15.267 ± 0.606, *p* < 0.0001) and ADHD HAI (15.78 ± 0.781, *p* < 0.0001) presented higher impulsiveness than the controls (10.35 ± 0.517) (Figure 5).

On motor impulsiveness, ANOVA also revealed an effect of group (F2,117 = 11.096, *p* < 0.0001). In this case, we also observed higher scores in ADHD LAI (14.733 ± 0.961, *p* < 0.01) and ADHD HAI (16.26 ± 0.938, *p* < 0.0001) when compared to the controls (10.525 ± 0.808) (Figure 5).

Similarly, ANOVA revealed an effect of group on non-planning impulsiveness (F2,117 = 8.869, *p* < 0.01). We observed higher scores in both ADHD LAI (15.767 ± 1.078, *p* < 0.05) and ADHD HAI (17.48 ± 0.988, *p* < 0.0001) than in the controls (12.325 ± 0.589) (Figure 5).

Finally, the ANOVA performed on total impulsiveness also indicated an effect of group (F2,117 = 19.827, *p* < 0.0001). In this case, we also observed higher values in the ADHD LAI (45.967 ± 1.842, *p* < 0.0001) and ADHD HAI (49.8 ± 2.178, *p* < 0.0001) than in the controls (33.65 ± 1.447) (Figure 5).

## 4. Discussion

Herein, we performed two different analyses on the same set of participants, assessing theta/beta ratio in subjects with ADHD symptomatology, either as one general group (Figure 2), or subdivided according to their performance in a working memory test (Figure 4). It should be noted that, while the Spanish version of the ASRS test has been validated both in Spain [35] and in a Mexican college population [36], and an impulsiveness test revealed notably higher scores in the groups with ADHD symptoms than in controls (Figure 3 and Figure 5), we did not perform a full ADHD diagnosis. Thus, we refer only to subjects with ADHD symptoms, and not to subjects with an ADHD diagnosis or ADHD patients. This approach has been previously used in other studies [37,38,39].

When analyzing all the subjects with ADHD symptoms, we found an increased theta/beta ratio at O1. Moreover, we observed a clear but non-significant tendency towards an increased theta/beta ratio at F4 (*p* = 0.0828), Fz (*p* = 0.0886), Cz (*p* = 0.0862) and O2 (*p* = 0.05) (Figure 2). The analysis of Barrat’s impulsiveness test revealed increased impulsiveness in all four subscales of the test (cognitive, motor, non-planning and total) in the ADHD group (Figure 3).

The analysis of their performance in the working memory test revealed no difference between the ADHD and the control groups (Figure 1A); however, there was high heterogeneity in the performance of the group with ADHD symptoms. It is known that ADHD presents high etiological diversity, as well as variation in the neurophysiological correlates of this disorder, in different studies [21,25,26,27]. Therefore, we assessed whether our ADHD subjects were indeed heterogeneous through their performance in a working memory test.

It should be noted that, in addition to the reported variation in ADHD etiology and brain correlates, there is at least another report revealing both cognitive and neurophysiological heterogeneity in a group of subjects with alcohol addiction. The study reports two subpopulations of alcohol-dependent subjects within the same study population. One of the groups presented increased delta and theta activity, while the other presented decreased delta and theta activity. Furthermore, both groups presented different results when the authors analyzed p300, as well as variations in cognitive performance [34]. Herein, we also found two subgroups within the subjects with ADHD symptoms, presenting different performance in the working memory test, as well as different neurophysiological correlates. Taken together, these reports highlight the inherent variability that can be found within a group of subjects sharing the symptomatology for a specific disorder.

As observed in Figure 1B, the accuracy index of the ADHD LAI in the working memory test was much lower than the one observed for the other two groups (*p* < 0.0001). Moreover, subjects in the ADHD HAI group performed even better than the controls (*p* < 0.05). This is to be expected, since we used the accuracy index to subdivide the ADHD group, leaving only those who scored above 0.9 in the ADHD HAI group. Conversely, in the control group we did not make this distinction, and included subjects who rated ≤ 0.9 as well as those with higher scores. The presence of subjects with lower scores in the working memory test in the control group could be due to the diversity commonly found in any population, and not to the presence of some pathological process in these subjects. Moreover, it should be noted that while there was a significant difference between the control and ADHD HAI groups (*p* < 0.05), the difference between both of these groups and the ADHD LAI group was much larger (*p* < 0.0001). It could be argued that the ADHD LAI group could also include subjects whose poor performance in the working memory test was not due to this disorder, but rather, is evidence of normal neuropsychological heterogeneity. This could be the case for some of the subjects, and cannot be disregarded with all the tests applied in this work; however, given that ADHD patients often present cognitive deficit [48,49], the number of subjects in the ADHD LAI group with a low accuracy index whose performance was not associated with ADHD symptomatology would be low.

The analysis of the theta/beta ratio considering the two subgroups of subjects with ADHD symptomatology (Figure 4) revealed increased scores in the ADHD LAI group when compared with the controls at Cz and O2. Moreover, ANOVA indicated a non-significant tendency for an effect of group at O1 (*p* = 0.0501) and T6 (*p* = 0.0724). Post hoc tests also indicated a higher theta/beta ratio in the ADHD LAI group than in controls for these derivations. It is noteworthy that theta/beta ratio for the ADHA HAI group did not differ from either the control or ADHD LAI groups.

Regarding Barrat’s impulsiveness test, both ADHD HAI and ADHA LAI presented higher impulsiveness than the controls in all four subscales (Figure 5). Furthermore, for motor and non-planning impulsiveness, the difference between the ADHD HAI and control groups was larger than between the ADHD LAI and control groups.

Regarding the areas where changes in theta/beta ratio were observed, they were mainly in central and occipital regions, with slight differences when analyzing one and two ADHD groups. This is in agreement with previous reports indicating an increase in theta/beta ratio at Cz in children with ADHD [2,28], and theta/beta at this site is often the target for neurofeedback treatments [50,51]. A previous report on theta/beta ratio in adult ADHD patients failed to find a clear change in this population [29], probably due to a heterogeneous composition of their ADHD group. It is noteworthy that while an increase in theta/beta ratio was observed in the unified ADHD group (Figure 2), once the subdivision was applied, it was observed only in the ADHD LAI group (Figure 4). The ADHD HAI group did not differ from the controls; however, it did not differ from the ADHD LAI either. It is possible that our subdivision did not allow for a complete distinction of the different subgroups present, yielding a still mixed group of subjects, and thus, intermediate performance in the working memory test.

It is difficult to determine the cause of the difference in brain activity observed in the two ADHD subgroups. An increased theta/beta ratio has been observed in conditions such as cognitive impairment in elder people [52,53], and in Lewy body disease [54]. Furthermore, a study on P300 and the theta/beta ratio suggests that it could be used as a marker of cognitive processing capacity [55]. Altogether, the current literature suggests that higher theta/beta ratios are associated with poorer cognitive performance. It is thus interesting that herein, we observed increased theta/beta ratios in the ADHA LAI group, but not in the ADHD HAI group. Moreover, the ADHA HAI group presented good performance in the working memory test, despite rating positive for ADHD in the ASRS-V1.1 test and presenting high impulsiveness scores in Barrat’s test (Figure 5). It should be noted that the participants in this study were university students of the health sciences area (mostly medicine students) with constant cognitive demand. Thus, it is plausible that most of the subjects within our ADHD groups have developed compensatory strategies to overcome ADHD symptoms in order to attain performance that allows them to continue with their studies. It is then possible that the absence of an increase in theta/beta ratio present in the ADHD HAI group, when compared with the controls, is the neurophysiological correlate of a compensatory mechanism in these subjects that allows them to achieve good performance in the working memory test, despite the presence of neurophysiological alterations leading to ADHD symptoms.

One of the current challenges in ADHD treatment is precisely the different effects that a given treatment might have on different subjects, which is probably due to the heterogeneity in the etiology and neurophysiological correlates of this disorder in different patients [21]. This makes it harder to decide, for example, whether a pharmacological treatment will be the best choice for a given patient, a decision that involves multiple factors and is not always a straightforward result of the patient’s symptoms [56].

Given the high heterogeneity of ADHD patients, there is increasing interest in personalizing treatment, either pharmacological or non-pharmacological, for each patient in order to attain the best possible therapeutic response with minimal adverse effects. Several approaches have been used to attain this goal through a better understanding of the mechanisms through which pharmacological treatments produce a reduction in ADHD symptoms. For example, a recent randomized control trial evaluated the effect of lisdexamfetamine on ADHD symptoms and brain structure through magnetic resonance imaging. Their results suggests that stimulant medication (such as amphetamines) produce an improvement in ADHD symptoms through a decrease in striatal and thalamic dynamic functional connectivity, thus increasing connection stability [57]. A complementary approach also under research is determining the neurophysiological changes produced by pharmacological treatment in patients with good or bad responses to this treatment. For example, a study performing quantitative analysis of EEGs of patients before and after treatment with methylphenidate reports an improvement in hyperactivity correlated with increased slow-wave activity, reduced fast-wave activity, and increased slow/fast ratios [58].

Other studies aim to identify specific neurophysiological features that could predict a good response to pharmacological treatment. For example, in children and adolescents, increased basal theta activity [59], and a high theta/alpha ratio [60], are associated with a good response to stimulant medication.

All the aforementioned studies provide important insights into both the neurophysiological mechanisms through which pharmacological treatments produce an improvement in ADHD symptoms, and possible strategies through which to improve treatment selection, through analysis of their effect on brain activity. However, these approaches need specific tests and equipment that are not always available. Our results suggest that cognitive performance could help us distinguish between different subgroups of ADHD patients, by means of an easy and affordable approach.

Among the strategies explored until now, neuropsychological profiling presents two important advantages as a tool for patient’s classification: (1) The distinction of different cognitive profiles provides information on brain processes affected differentially in persons with the same ADHD diagnosis, probably reflecting more accurately the main changes in brain function in each subgroup. (2) Neuropsychological profiling could allow for the inexpensive screening of patients to determine the specific subgroup to which they belong. Further research is needed to fully understand the scope of the cognitive profiling of ADHD patients as a strategy to classify them, in order to select the most suitable therapeutic approach for each group of patients. It would be necessary to know both the cognitive performance and neurophysiological correlates of each sub-group. However, given the low cost of this procedure, this knowledge could provide an accessible option to guide treatment selection.

Many of the subjects with ADHD symptoms in our study also rated positive for other conditions, such as alcohol use disorders, depression and suicide risk. While comorbidities make it harder to define specific subgroups of subjects with ADHD symptoms, it is worth noting that it is quite frequent for ADHD patients to present comorbidities [46,47], which, in combination with the variability in ADHD etiology, might produce higher variability in brain electrical activity within the same group of subjects sharing similar symptomatology for ADHD. This combination will certainly affect other brain functions including cognitive performance, a function also affected in other conditions related to dopaminergic system dysfunction [61]. Thus, neuropsychological profiling could prove a useful aid to sub-classify ADHD patients through the effect of their condition on brain function. As the neuropsychological profile becomes wider, this classification will become more specific, allowing for close follow-up of the effects of treatments, thus leading to the identification of the most suitable treatment for each patient.

The prevalence of ADHD symptoms among the participants in this study is higher than the reported community prevalence of ADHD, which is around 5% for children and even lower for adolescents [62]. Several reasons exist for this apparent discrepancy. First, we were not assessing ADHD prevalence in our population, but rather, studying cognitive performance and neurophysiological correlates in those participants who rated positive for ADHD symptoms. Thus, we were not sampling a specific number of participants randomly selected from our population, but rather, inviting students to participate and incorporating information from those who were interested in taking part in the study. Second, when inviting students to participate in the study, we explained to different groups of students the objective of the study, including our interest in determining neurophysiological correlates and cognitive performance in subjects with ADHD symptoms, in an attempt to better understand this condition. Thus, it is natural that those students who felt inattentive or impulsive were more prone to participate in the study. These two arguments imply an important bias in the incorporation of participants in the study, rendering it unreliable for prevalence determination, albeit robust for the purpose we intended.

A limitation of this study is that we categorized our subjects with ADHD symptoms based on a single test (working memory), probably disregarding other cognitive features that might prove useful to distinguish subpopulations among subjects with ADHD symptoms. Our selection of working memory performance to classify subjects with ADHD symptoms was due to the large variety of conditions in which working memory is affected, including ADHD [63], multiple sclerosis [64], substance use disorders [65], depression [66] schizophrenia [67] and obsessive–compulsive disorder [68]. The variety of conditions in which working memory is impaired suggests that this cognitive skill can be affected at different levels, where different alterations in brain activity might lead to different alterations in cognitive skills. Thus, alterations in working memory might prove sensitive to different changes in brain circuits, although more than one of these variations in brain activity could lead to the same behavioral symptoms (ADHD in our study).

However, even though we used only one test to classify the subjects, we already found that our population with ADHD symptoms was not homogeneous, differing at least in their performance in the working memory test and in their theta/beta ratios. It is probable that a wider neuropsychological assessment would lead to a better classification of ADHD subjects. For this purpose, the inclusion of other tests for attention and impulse control, such as CPT or the Iowa gambling test, could prove useful in future studies. In order to achieve this goal, further research should be conducted on three topics: (1) the neuropsychological profiling of ADHD patient subgroups; (2) determination of the neurophysiological correlates of each ADHD subgroup; and (3) a close follow-up of the effects of selected treatments on ADHD symptoms in each subgroup.

## 5. Conclusions

Subjects with ADHD symptoms present high variation in brain activity, as well as in their response to treatment. Previous studies suggest the existence of different subgroups of ADHD patients with different etiologies for the disorder and different responses to treatments. In order to achieve more optimal treatment selection, strategies designed to identify these subgroups will allow for the development of therapeutic strategies designed for each specific subgroup of patients.

There are articles reporting the classification of ADHD patients based on their qEEG [25,26,27]. Our work suggests that different neurophysiological correlates also imply different cognitive affectation in ADHD patients. Robust neuropsychological profiling matching specific neurophysiological profiles in ADHD patients still needs further research; however, it is an attainable goal. From a clinical approach, robust neuropsychological profiling matching specific brain activity profiles in ADHD patients will allow for an easy, rapid and affordable tool for patient subgrouping and treatment selection, leading to a better therapeutic effect and fewer adverse reactions.

## Figures and Tables

**Table 1:** Age and gender compositions of the groups used for the first analysis. F, females; M, males.

	Total (Age ± SEM)	F (Age ± SEM)	M (Age ± SEM)
Controls	66 (19.112 ± 0.176)	36 (19 ± 0.14)	30 (19.245 ± 0.351)
ADHD	80 (19.387 ± 0.199)	49 (19.495 ± 0.247)	31 (19.216 ± 0.337)

**Table 2:** Age and gender compositions of the groups used for the second analysis (two sub-groups of subjects with ADHD symptoms). F, females; M, males.

	Total (Age ± SEM)	F (Age ± SEM)	M (Age ± SEM)
Controls	40 (19.297 ± 0.272)	20 (19.128 ± 0.189)	20 (19.465 ± 0.514)
ADHD LAI	30 (19.112 ± 0.19)	19 (19.207 ± 0.244)	11 (18.949 ± 0.308)
ADHD HAI	50 (19.552 ± 0.297)	30 (19.678 ± 0.373)	20 (19.362 ± 0.498)

ADHD LAI, subjects with ADHD symptoms and low accuracy index in the working memory test; ADHD HAI, subjects with ADHD symptoms and high accuracy index in the working memory test.

## Data Availability

The data presented in this study are available on request from the corresponding author. The data are not publicly available due to privacy restrictions.
